# *Bacillus cereus* in food safety: a bibliometric analysis

**DOI:** 10.3389/fmicb.2025.1574802

**Published:** 2025-06-04

**Authors:** Muhammad-Ehtesham Abdul, Enrico Pavoni

**Affiliations:** Istituto Zooprofilattico Sperimentale della Lombarda e dell’Emilia Romagna, “Bruno Ubertini”, Brescia, Italy

**Keywords:** bibliometric analysis, foodborne pathogens, cereulide, antimicrobial resistance, VOSviewer

## Abstract

*Bacillus cereus*, a spore-forming pathogen, poses significant food safety risks due to its prevalence in diverse food matrices and ability to produce emetic and diarrheal toxins. This study presents the first bibliometric analysis of global research on *B. cereus* in food safety, examining 898 Scopus-indexed articles (2000–2024). Data were extracted using the search query “*Bacillus cereus*” OR “*B. cereus*” AND “Food Safety” in titles/abstracts, followed by quantitative and visual analyses via VOSviewer and the bibliometrix R-package. Metrics included annual growth rates, citation trends, country/institution contributions, and keyword co-occurrence. Collaborative networks and author productivity were mapped using co-authorship analysis. Results revealed an 8.29% annual publication growth, with China (38.86%), South Korea (22.05%), and the United States (18.26%) as leading contributors. Citation analysis highlighted seminal works on pathogenicity (e.g., enterotoxins, antimicrobial resistance), while keyword co-occurrence identified emerging themes such as virulence genes, cereulide, and sustainable mitigation strategies (e.g., probiotics, bacteriocins). Critical gaps persist in understanding *B. cereus* behavior in novel food matrices (e.g., plant-based alternatives) and the efficacy of emerging preservation technologies. This analysis underscores the need for interdisciplinary approaches integrating genomics, food science, and public health to address risks in global supply chains. The findings provide a roadmap for future research, advocating for advanced surveillance, innovative interventions, and policy refinement to combat this resilient pathogen.

## 1 Introduction

*Bacillus cereus* is a Gram-positive, spore-forming, and facultative anaerobic rod ubiquitously found in the environment (Jessberger et al., 2020). Hence, vegetative cells—and especially spores—can easily enter the food chain. Consequently, *B. cereus* is detected in a wide variety of foods, such as starchy foods and rice, raw and pureed and minimally processed vegetables, bread, milk, and dairy products, meat products, and ready-to-eat foods (Andersen Borge et al., 2001; Carlin, 2011; Johnson, 1984; Rahnama et al., 2023). This organism can cause foodborne illness in humans by releasing an emetic toxin directly into food products that support its growth, or by producing a diarrheal enterotoxin in the consumer’s gut after ingesting *B. cereus*-contaminated food (Bottone, 2010; Stenfors Arnesen et al., 2008). These characteristics make *B. cereus* an important concern in food safety (Rahnama et al., 2023). *B. cereus* was responsible for approximately 1.4 to 12% of global foodborne illness outbreaks. In 2022, the European Food Safety Authority (EFSA) and the European Centre for Disease Prevention and Control (ECDC) reported approximately 5,763 foodborne outbreaks across the EU, representing a notable 44% increase compared to 2021. Of these, 306 outbreaks were caused by *B. cereus* (Monitoring of Foodborne Diseases | EFSA, 2023). In the United States, 1.74% of foodborne outbreaks were reported between 1998 and 2008 as related to *B. cereus* (Bennett et al., 2013).

A bibliometric analysis is a quantitative research method that uses statistical techniques to analyze and evaluate scientific publications (Ellegaard and Wallin, 2015). This approach enables the identification of key authors, institutions, and research trends within a field. Researchers can uncover significant research gaps, emerging topics, and influential studies by analyzing publication patterns and citation networks (Yan et al., 2024). Numerous bibliometric studies have been published focusing on specific pathogenic bacteria, gastrointestinal diseases, and zoonotic infections (Şahin et al., 2021; Şahinoğlu and Alkan, 2024; Sweileh, 2021; Yan et al., 2024). Additionally, many bibliometric studies examine various aspects of food safety (Bouzembrak et al., 2019; Shen et al., 2021; Wahyuni et al., 2019). However, none was carried out about the role of *B. cereus* in food safety. Therefore, we conducted this bibliometric analysis to evaluate global research productivity on *B. cereus* in the field of food safety and to present an overview of current *B. cereus* research, aiming to identify emerging trends and potential directions for future studies.

## 2 Methods

### 2.1 Data collection

**Figure 1:**
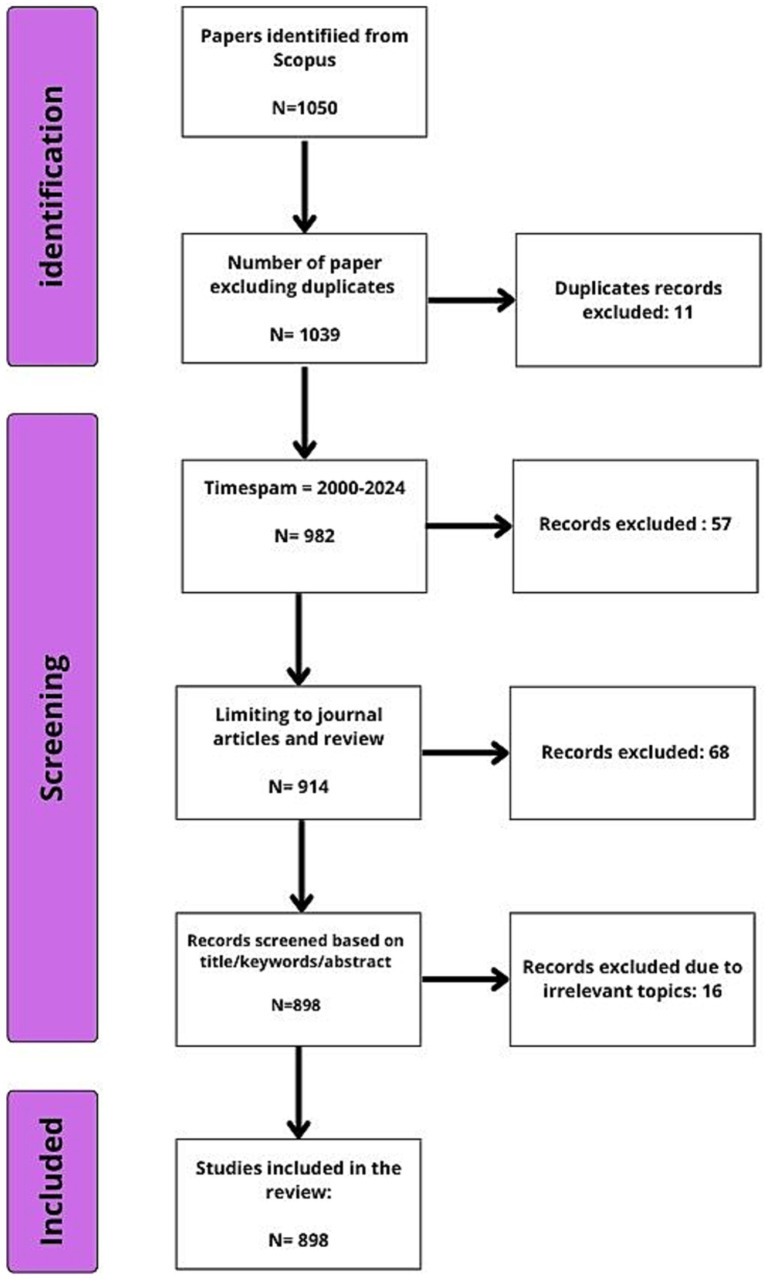
PRISMA flowchart: study selection process for articles on *Bacillus cereus* in food safety.

For the purpose of this study data were collected from articles published in Scopus index journals.^1^ The search query used was “*Bacillus cereus*” OR “*B. cereus*” AND “Food Safety”; keywords were used in the article title and abstract search. The search query was limited to articles and reviews published between 2000 and 2024. No articles were excluded based on language. According to Scopus policy, all published articles indexed in Scopus must include an English abstract. Consequently, even articles not written in English have an English title and abstract, facilitating the content analysis. Figure 1 presents a schematic outline of the steps followed to retrieve data for this study.

### 2.2 Data analysis

Bibliometric analyses and network visualizations were performed using VOSviewer version 1.6.16 software (van Eck and Waltman, 2013) and bibliometrix R-package, an open-source tool for executing a comprehensive science mapping analysis of scientific literature (Aria and Cuccurullo, 2017). VOSviewer offers various display options for maps, along with functionalities such as zooming, scrolling, and searching. The most commonly created maps using this software include co-authorship, keyword co-occurrence, citation, bibliographic coupling, and co-citation maps, all based on bibliographic data (van Eck and Waltman, 2009).

## 3 Results

### 3.1 Descriptive analysis

#### 3.1.1 Main information

The search query retrieved a total of 898 articles on *B. cereus* in food safety, published between 2000 and 2024 (Figure 1). Among these, 843 articles (93.84%) were written in English, while the remaining were published in other languages, primarily Chinese (43 articles, 4.78%) and Korean (10 articles, 1.11%). These publications were authored by 4,336 researchers across 343 distinct sources (e.g., journals, books) from 69 countries. Of the total articles, 798 (88.86%) were research articles, and the remaining 99 (11.14%) were review articles.

#### 3.1.2 Yearly distribution and growth trend of publications

**Figure 2:**
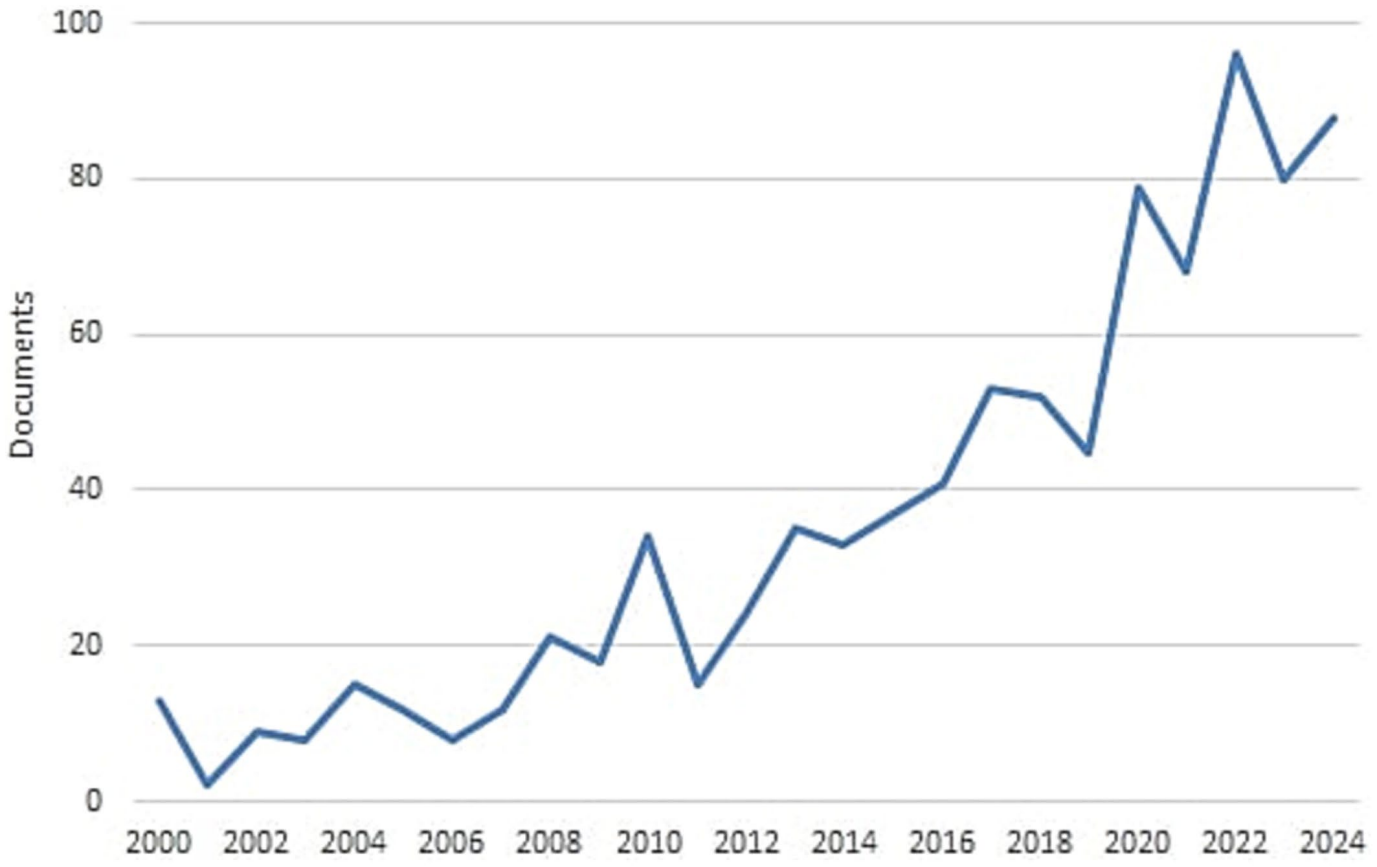
Total number of publications for year.

Figure 2 shows the annual number of publications from 2000 to 2024. This shows a significant increase in this field of study reaching a maximum of 96 articles in 2022. Regarding the considered period of time, the annual growth rate (AGR) is 8.29%. This metric assesses how rapidly the volume of publications has grown (or declined) over the time, providing insight into the development trend within the research field (Bornmann and Mutz, 2015). A positive AGR indicates a year-over-year increase in the number of publications, suggesting that the field or topic is expanding rapidly, reflecting either rising interest, increased funding, or higher research activity over the time. This means that each year there has been an approximate 8.29% increase in the number of publications, indicating strong momentum within this research area. The Document Average Age (DAA) provides a measure of the recency of the literature analyzed, indicating whether the publications in the dataset tend to be more current or older. A DAA of 6.95 years suggests that the field under study is relatively recent but not exclusively a cutting-edge, as it includes a balanced mix of both recent and moderately older publications. This metric helps contextualize the dataset within the research timeline, offering insight into the age distribution of the literature reviewed.

#### 3.1.3 Citation analysis

The 898 documents received a total of 23,865 citations, resulting in an average citation per publication of 26.57. The retrieved articles have an h-index of 79, indicating that 79 of these articles have been cited each at least 79 times—a figure that may increase over time. The average annual citations per article are detailed in Table 1, showing the highest values for older articles (e.g., 112.33 for 2002) and the lowest for recent articles (e.g., 6.69 for 2023). Considering the aging of references in this field, the Price’s index (de Price, 1969) was calculated as follows


$$\text{Price}\prime s\;\text{index}=\frac{\text{number of citations less than}\;5\;\text{year}\;\text{old}}{\text{total number of citations}}\times 100\%$$

**Table 1:** Annual number of publications and citation on *B. cereus* in food safety.

Year	Number of publications	%*N* = 898	TC	C/A
2000	13	1.45	1,012	77.85
2001	2	0.22	140	70
2002	9	1.00	1,011	112.33
2003	8	0.89	345	43.12
2004	15	1.67	794	52.93
2005	12	1.34	730	60.83
2006	8	0.89	248	31
2007	12	1.34	395	32.92
2008	21	2.34	706	33.62
2009	18	2.00	604	33.56
2010	34	3.79	1,154	33.94
2011	15	1.67	513	34.2
2012	24	2.67	525	21.88
2013	35	3.90	1,444	41.26
2014	33	3.67	1,511	45.79
2015	37	4.12	1,157	31.27
2016	41	4.57	1,254	30.59
2017	53	5.90	2,582	48.72
2018	52	5.79	1981	38.1
2019	45	5.01	1,091	24.24
2020	79	8.80	1826	23.11
2021	68	7.57	1,384	20.35
2022	96	10.69	865	9.01
2023	80	8.91	535	6.69
2024	88	9.80	58	0.66

TC, total citations; C/A number of citations per article.

The Price’s index of the references shown in Table 1 is 19.55%, which means that 19.55% of them were from the last 5 years, indicating that the aging of publications is relatively slow. The top cited articles were mostly about foodborne pathogens and antimicrobial activity. The most cited article is “Foodborne pathogens” (Bintsis, 2017) published in *AIMS Microbiology* which has 565 citations.

#### 3.1.4 Country-based analysis of publication contributions

**Figure 3:**
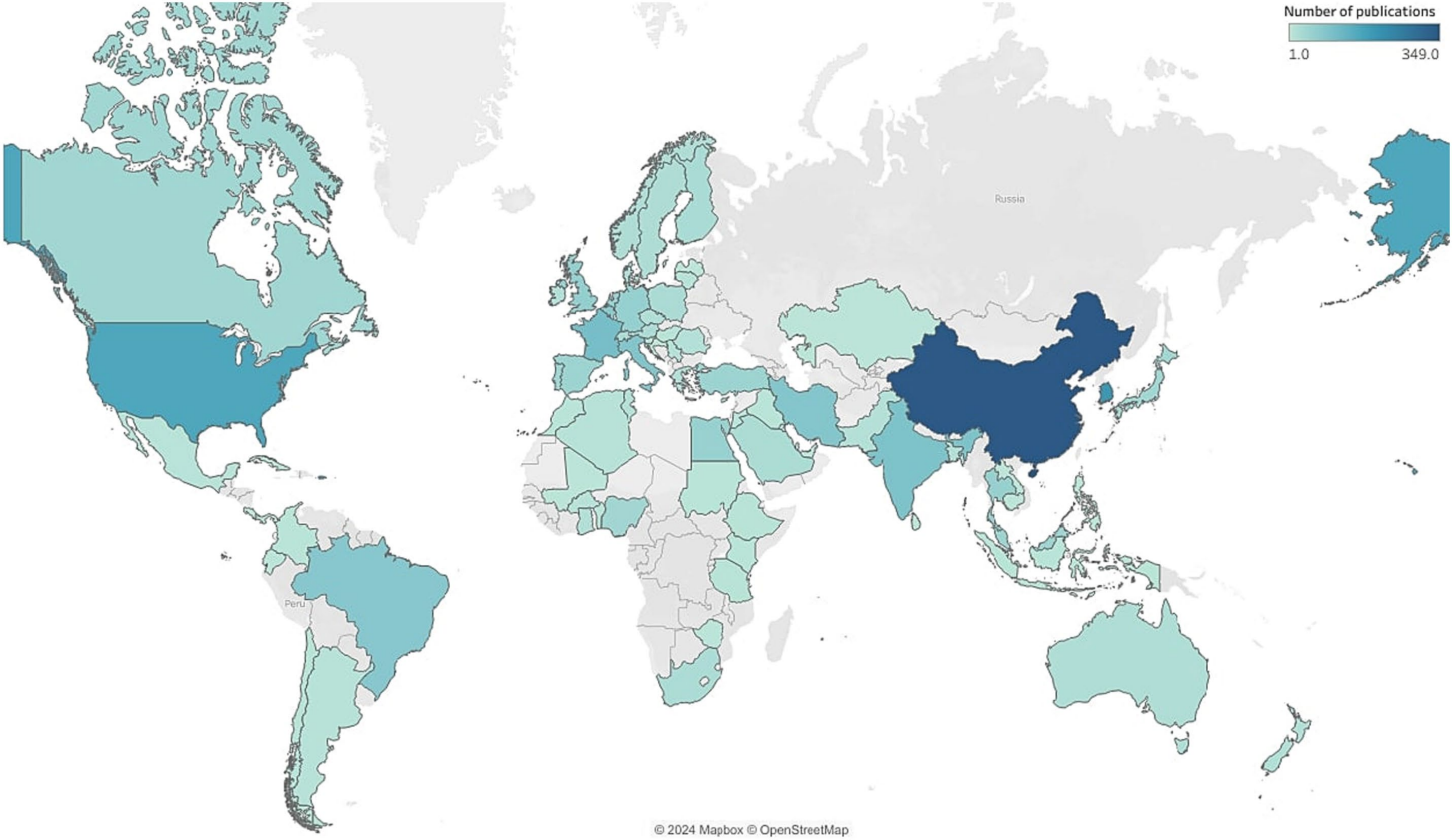
Country based scientific production (Map created using Mapbox©).

**Figure 4:**
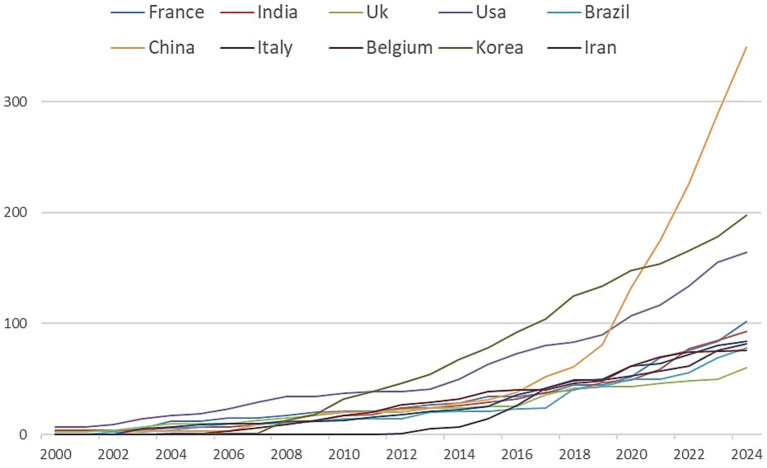
Country-based scientific production over the time.

The 898 articles in this dataset originated from 68 countries (Figure 3). Among these, China is the main contributor with 349 publications (38.86%), followed by South Korea with 198 articles (22.04%) and the United States with 164 (18.26%). France and India follow with 102 (11.35%) and 93 (10.35%) publications, respectively. The other countries in the top 10 for publication count are detailed in Table 2. It is important to note that the percentages reported in Table 2 exceed 100% when summed because they represent the relative contribution of each country to the total publications, accounting for overlapping contributions due to international collaborations. For example, a single article with authors from both China and South Korea would be counted under both countries’ contributions. This methodology reflects the collaborative nature of research and ensures that all contributions are appropriately represented. A cumulative count of contributions by country on the original 898 articles results in 2,318 publications, indicating a substantial international collaboration. The United States ranks as the top collaborator, participating in 27 joint publications, followed by the United Kingdom (22 citations), China and France (both with 21), and finally Italy and Belgium with 20 and 18 publications, respectively. The United States also leads in citations, with 2,530, followed by China (2,356), South Korea (2,056), Belgium (1,359), and Greece (879). Greece holds the highest average citation count per article, with an average of 125.6. An analysis of publication trends over time (Figure 4) reveals a rapid increase in scientific output from China, the United States, and South Korea over the past decade, while European countries such as France and Italy have shown more modest growth.

**Table 2:** Top 10 countries in number of publications.

Country	Number of publications	%*N* = 898	C/A
China	349	38.86	18.1
South Korea	198	22.05	25.7
United states	164	18.26	46
France	102	11.36	25.6
India	93	10.36	22
Italy	84	9.35	22.4
Iran	82	9.13	13.1
Brazil	78	8.69	30
Belgium	76	8.46	48.5
United Kingdom	60	6.68	43.7

C/A number of citations per article.

#### 3.1.5 Authors, institutions, and sources analysis

The leading institutions by publication count are listed in Table 3, with Chinese institutions as the most prolific contributing total 42 publications. Ghent University and Universiti Putra Malaysia, resulted as the most productive, with 32 and 28 articles respectively, if individually considered. Other European or American affiliations seem to have not much contributed if compared to Table 3. A cumulative analysis of contributions by institutions indicates a total of 2,220 publications, which exceeds the initial count of 898 articles. This discrepancy underscores the extensive collaboration among institutions in this field. Table 4 highlights the top 10 high-productivity authors, providing an overview of the most prolific and cited researchers. The distribution of publications among the authors was analyzed using Lotka’s Law which, in bibliometric studies, posits that the number of authors contributing n publications is inversely proportional to $n^{2}\;$ (Coile, 1977). This analytical framework provides insights into the degree of inequality in scientific outputs, highlighting the prevalence of a small group of highly prolific authors alongside a larger group of less productive contributors. In this study, the productivity of authors was assessed to determine whether the observed distribution aligns with Lotka’s theoretical model. Statistical validation, including goodness-of-fit tests, was performed to evaluate the conformity of the dataset to Lotka’s prediction. The Lotka’s prediction was calculated using the following formula:


$$f(x)=\frac{C}{x^{n}}$$

$$C=\frac{1}{\sum_{x=1}^{\infty }\frac{1}{x^{n}}}$$

**Table 3:** Top ten productive institutions in the field.

Affiliation	Number of publications	%*N* = 898	Country
Ghent University	32	3.56	Belgium
Universiti Putra Malaysia	28	3.12	Malaysia
Islamic Azad University	16	1.78	Iran
Zhejiang University	15	1.67	China
South China Agricultural University	14	1.56	China
Wageningen University	14	1.56	Netherlands
Yeungnam University	14	1.56	South Korea
China Agricultural University	13	1.45	China
Korea Food Research Institute	13	1.45	South Korea

**Table 4:** Top ten productive authors in publishing articles on *B. cereus* in Food Safety.

Affiliation	Number of publications	C/A	Country
Ehling-Schulz M.	11	39.64	Austria
Uyttendaele M.	10	46.40	Belgium
Zwietering M.	10	36.20	Netherlands
Rajkovic A.	9	56.44	Belgium
Oh D.	8	31.75	Sud Korea
Abee T.	7	51.14	Netherlands
Ding Y.	6	56.00	China
Friedman M.	6	82.50	USA
Granum P. E.	6	35.17	Norway

C/A number of citations per article.

f(x): The proportion or count of authors producing x publications (x= 898).

n: Typically set to 2 in Lotka’s original formulation.

C: normalization constant.

**Figure 5:**
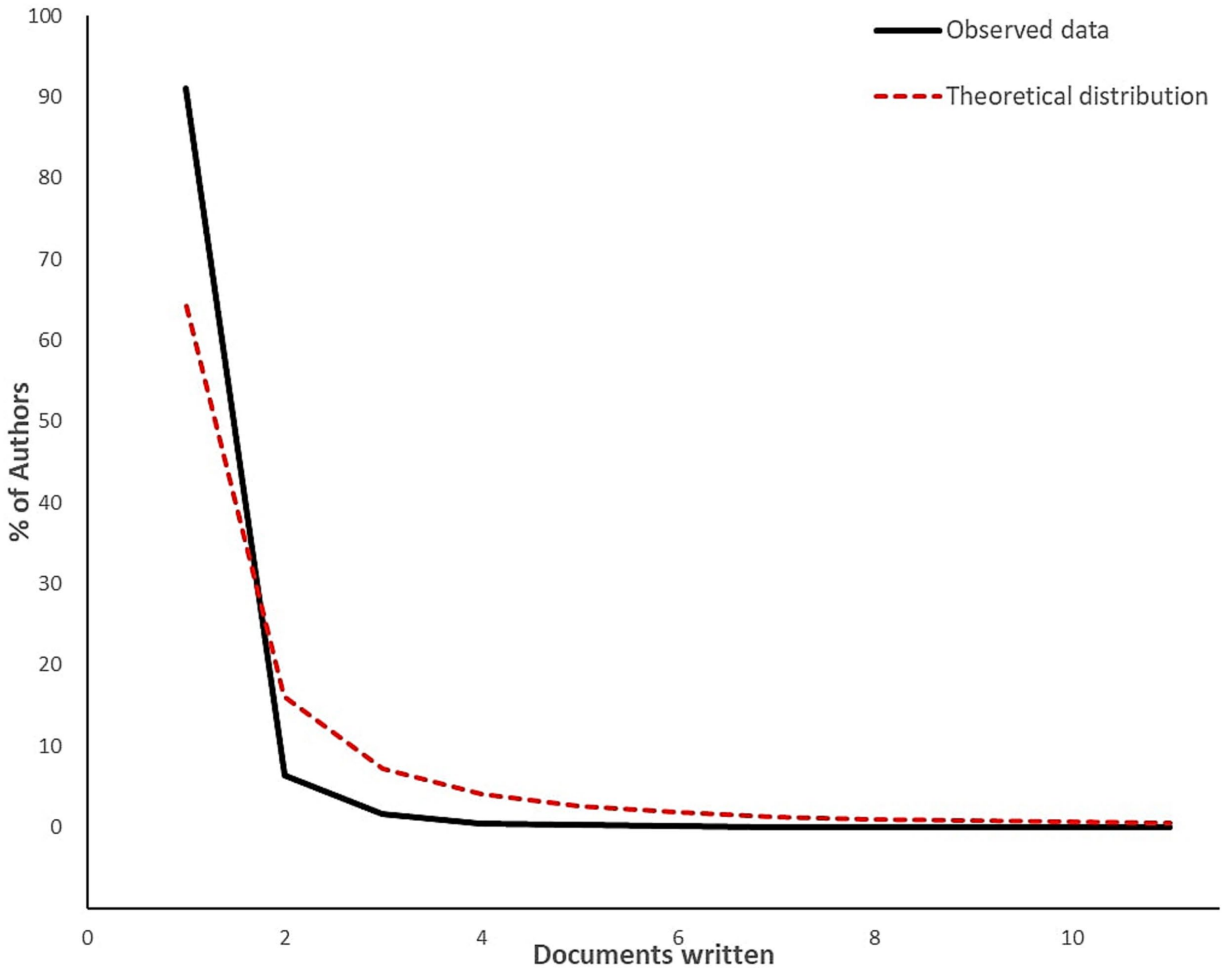
Author productivity through Lotka’s Law.

Figure 5 presents a visual comparison between the observed distribution of author productivity and the theoretical distribution described by Lotka’s Law, highlighting the areas of alignment and deviation. The most active journal in the field is the International Journal of Food Microbiology, which published 58 articles, followed by the Journal of Food Protection with 43 articles, as shown in Table 5. All journals in the top 10 list are indexed with an impact factor (IF), ranging from 1.9 to 6.0. Most of these journals are classified within the Q1 Journal Impact Factor (JIF) quartile. Furthermore, all journals in the top 10 are closely associated with food science and microbiology disciplines.

**Table 5:** Top ten productive journals publishing on *B. cereus* in Food Safety.

Journal	Number of publications	C/A	JCI	IF	JIF Quartile
International Journal of Food Microbiology	58	44.83	1.23	5	Q1
Journal of Food Protection	43	30.53	0.47	2.1	Q3
Food Control	40	30.53	1.31	5.6	Q1
Frontiers in Microbiology	33	38.48	0.89	4	Q2
Foodborne Pathogens and Disease	27	47.00	0.46	1.9	Q3
Journal of Applied Microbiology	21	36.33	0.67	3.2	Q2
Foods	18	13.11	1	4.7	Q1
Lwt-Food Science and Technology	18	21.44	1.34	6	Q1
Food Microbiology	17	55.06	1.17	4.5	Q1

JCI, Journal Citation Indicator; IF impact factor; JIF, quartile Journal impact factor quartile; C/A number of citations per article.

### 3.2 Visual analysis

#### 3.2.1 Cooperative network analysis

**Figure 6:**
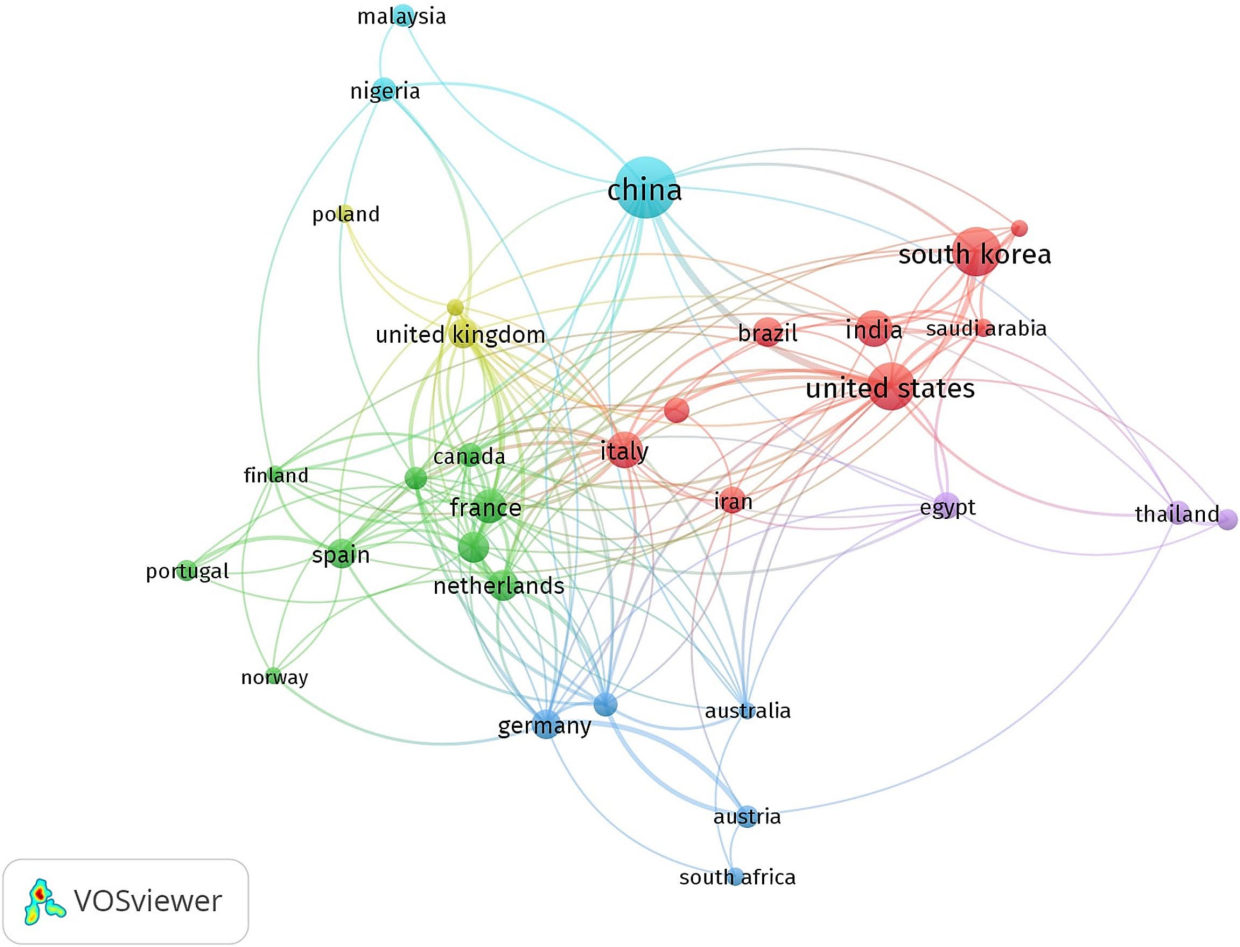
National collaboration networks.

There are primarily three categories of collaboration networks: between countries, between institutions, and between individual researchers. For this study, we utilized VOSviewer’s co-authorship feature to generate a national collaborative network, illustrated in Figure 6, with a threshold of 10 publications and 50 citations. In the diagram, node size corresponds to the volume of papers produced by each country, while connecting lines indicate international collaborations. Different colors are used to denote distinct groups, and the proximity of nodes reflects the similarity of research topics. Examining cooperation networks among nations reveals distinct groups connected by geography and scientific endeavors. European and Asian countries generally show strong interactions, forming solid collaborative clusters. China emerges as a key player in the network, with India and Japan also connected but demonstrating less intense cooperation compared to China. European nations, notably, display close interconnection. Italy France, and Germany form a robust group, characterized by a strong collaborative network encompassing both publication exchanges and reciprocal citations. The United States and Canada also hold central positions in the network, exhibiting intense scientific activity. Other countries, particularly in the Middle East and Western Asia, while having lower participation in the global cooperation network, still contribute to significant alliances(?) in this research area. Saudi Arabia and Iran, despite their relatively lower scientific output, are integrated into a network of international collaborations. Lastly, developing nations such as Egypt, Thailand, and Malaysia show more peripheral cooperation positions, but remain part of global networks, indicating their increasing involvement in international scientific dynamics.

**Figure 7:**
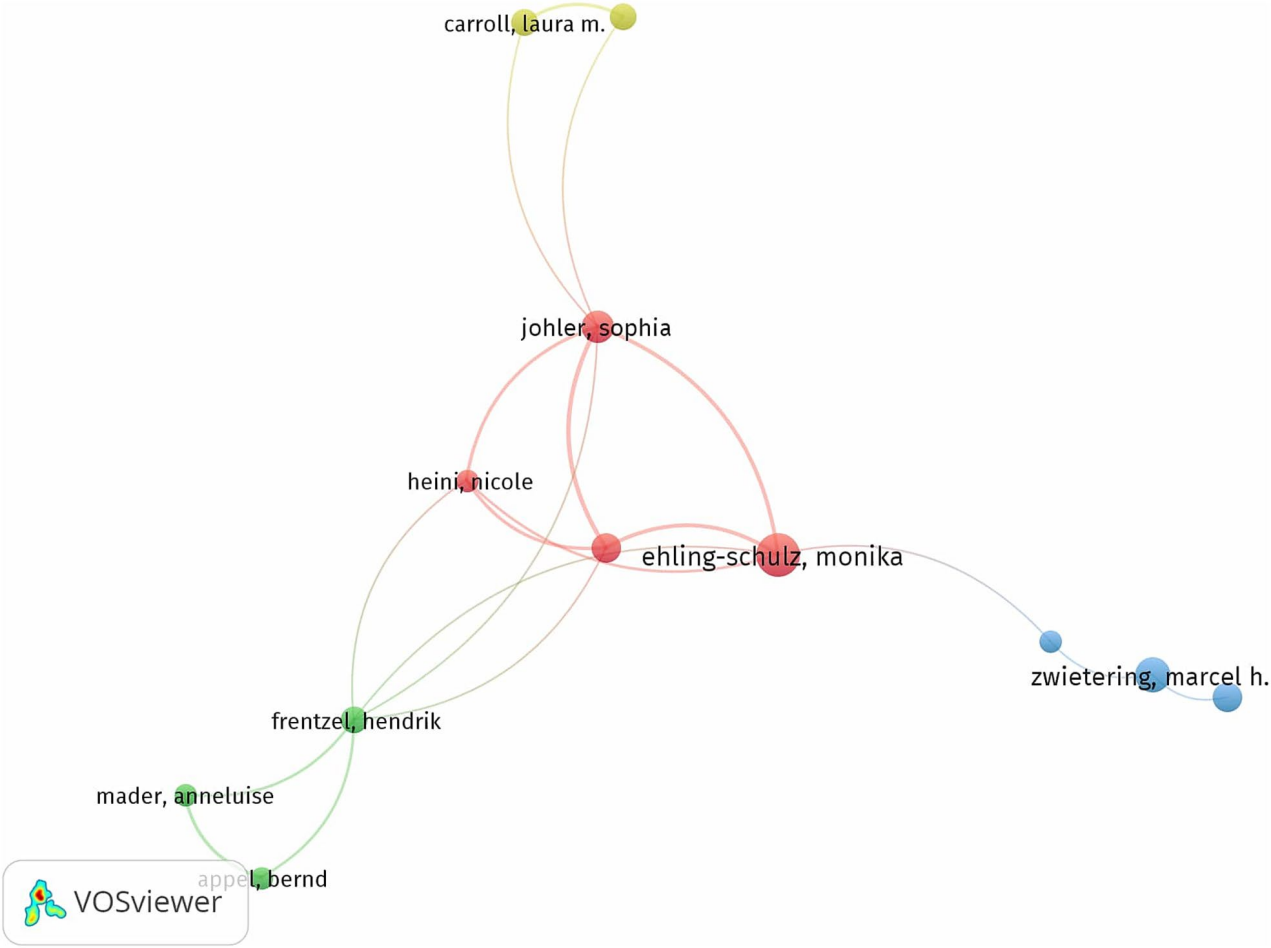
Authors cooperation networks.

The diagram illustrating the authors’ collaboration network effectively portrays the structure of the research teams. The intensity of collaboration among these teams can be assessed based on the proximity between the groups and the density of their connection lines. The co-authorship function in VOSviewer sets a minimum of two publications and at least 10 citations in order to generate the collaboration network (Figure 7). The cooperative network analysis conducted using VOSviewer highlights the collaborative relationships among key researchers in the field. The results reveal the existence of distinct research clusters, indicating the formation of collaborative groups based on shared interests. These clusters demonstrate varying levels of interconnectivity, with some groups showing strong collaborative links and others reflecting more isolated interactions.

In general, there is a dynamic structure of research cooperation, where some authors exhibit significant influence through a large number of collaborations and publications, while those remaining, despite fewer direct connections, have an important impact on the citation network. The collaborative patterns also suggest that certain researchers are pivotal to the development of specific issues, while others are more peripheral, contributing to a broader range of topics.

The network further reveals a temporal evolution, where some researchers focused their efforts on recent publications, and others have a more extensive history of collaboration and citation impact. This evolution reflects the nature of research priorities, with new themes gaining momentum and attracting more citations. Overall, the cooperative network analysis provides valuable insights into how collaborations influence the development of the research fields.

#### 3.2.2 Keywords co-occurrence analysis

**Figure 8:**
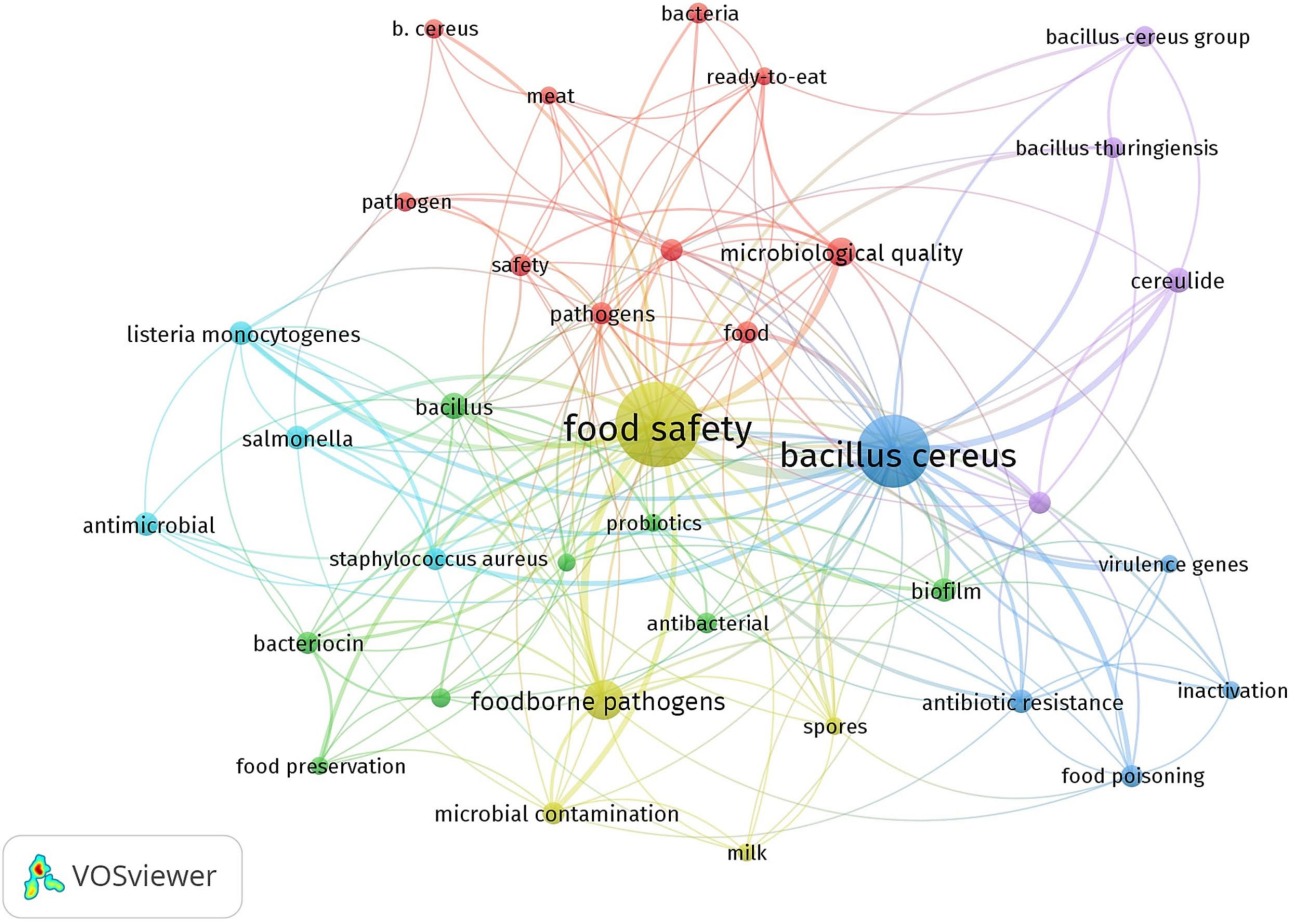
Co-occurence network for *B. cereus* in food safety keywords.

**Figure 9:**
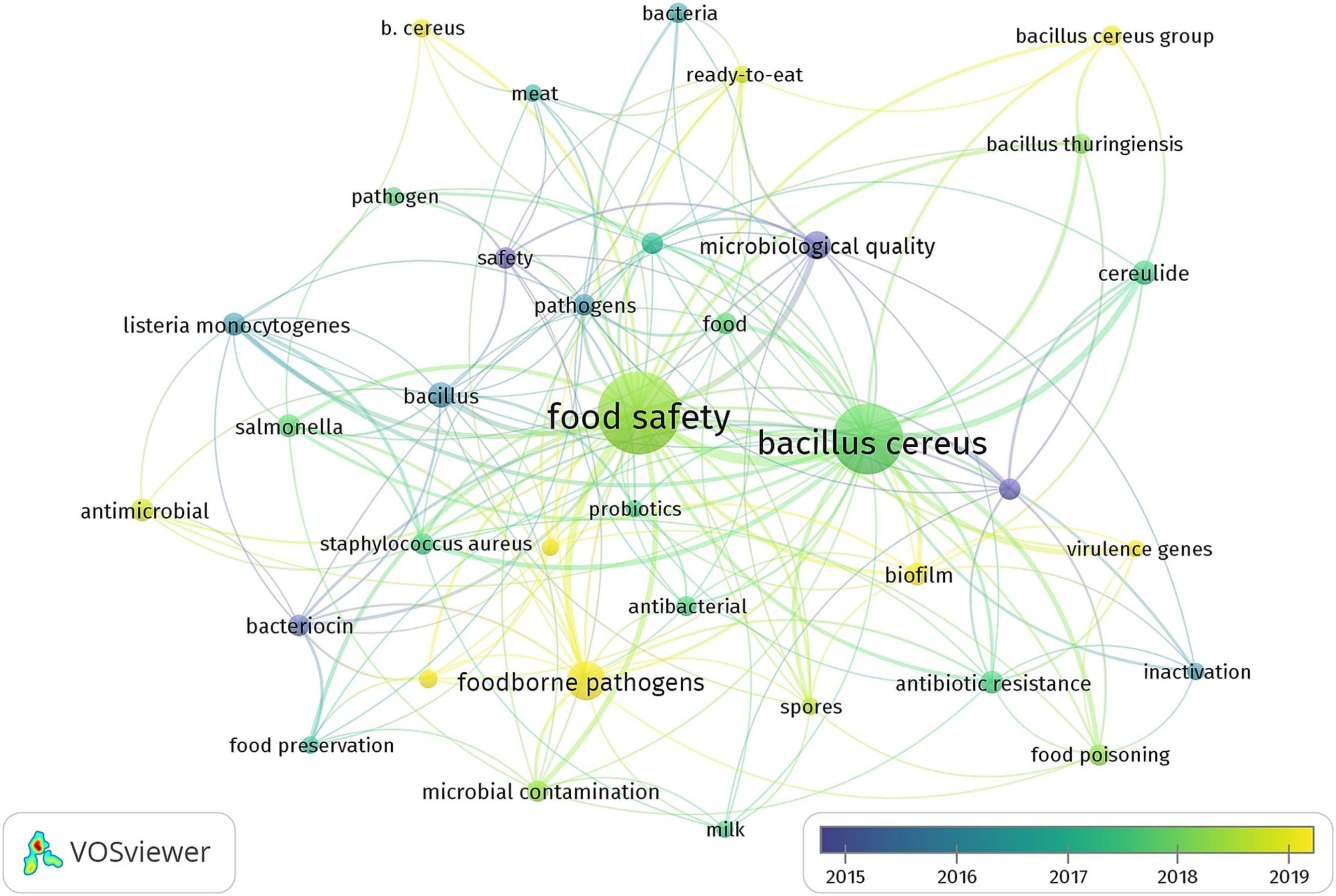
Co-occurrence overlay visualization for *B. cereus* in food safety keywords over time.

Keywords offer essential insights into the article’s content and the simultaneous presence of two or more keywords in a single paper is referred to as keyword co-occurrence (Su and Lee, 2010). The analysis of keyword co-occurrence enables the identification of research hotspots and helps track the evolution of research frontiers within a scientific domain (Lee and Su, 2010). In this study we utilized the co-occurrence analysis feature in VOSviewer applying a minimum threshold of 10 occurrences keywords for author to ensure meaningful and reliable results (Figure 8). The co-occurrence analysis of keywords highlights key themes and interconnected topics within the research topic of *B. cereus* and food safety. As expected *B. cereus* emerges as a central node with strong links (shown by the thickness of connecting lines) to related terms such as *B. cereus group* and *Bacillus thuringiensis* emphasizing the focus on this bacterium as one of the major concerns in food microbiology. The frequent association with keywords like *food safety foodborne pathogens* and *microbial contamination* underscores its significance as a foodborne pathogen of public interest and the impact on different points of the food chain. Research on *B. cereus* often explores its pathogenic mechanisms as evidenced by the prominence of terms like *enterotoxin cereulide* and *virulence genes*. Furthermore *B. cereus* is frequently studied alongside with other pathogens such as *Listeria monocytogenes* and *Salmonella* reflecting integrated efforts to address microbial risks in the context of foodborne diseases as a whole. Keywords such as *milk meat ready-to-eat* and *microbiological quality* suggest the diversity of food matrices examined for *B. cereus.* is These terms indicate an emphasis on perishable products and ready-to-eat foods that are suitable substrates for *B. cereus* proliferation and pose high risks of contamination and transmission to consumers. The term *food safety* acts as a critical hub illustrating the centrality of this topic in protecting public health through improved food handling processing and storage practices. Recent trends also point to an increased interest in mitigation strategies and alternative control measures. Keywords such as *probiotics lactic acid bacteria antimicrobial* and *bacteriocin* highlight research towards natural and sustainable solutions to prevent contamination and proliferation of *B. cereus* in food products. Similarly terms like *inactivation* suggest ongoing exploration of novel preservation and decontamination techniques aimed at enhancing food safety. The temporal distribution of keywords (Figure 9) reveals emerging research hotspots such as *B. cereus group antimicrobial activity* and *virulence genes* reflecting advances in molecular and genomic studies that have significantly contributed to a deeper understanding of the bacterium’s behavior and pathogenicity. Earlier studies predominantly focused on foundational aspects of *B. cereus* biology such as its taxonomy toxin production and role in foodborne illnesses. Keywords such as *enterotoxin cereulide* and *food poisoning* dominated earlier years emphasizing the identification and characterization of its virulence factors and public health implications. In contrast more recent research trends indicate a shift towards understanding the bacterium’s adaptability and resistance mechanisms as evidenced by the increased occurrence of terms like *antibiotic resistance* and *antimicrobial activity*. This suggests growing concern over the role of *B. cereus* in the broader context of antimicrobial resistance a critical global health challenge. Similarly keywords such as *virulence genes* and *genomics* point to the application of advanced molecular tools to uncover genetic determinants of pathogenicity and explore their variability across strains.

## 4 Discussion

The study of *B. cereus* in the context of food safety has undergone significant development over recent decades, with research evolving from basic bacteriology to more advanced molecular and genomic studies. This bibliometric analysis has offered a comprehensive overview of the scientific landscape, shedding light on publication trends, national and researcher collaboration networks, as well as the key thematic areas driving the field. In terms of publication trends, it is evident that the focus of *B. cereus* research has shifted in response to emerging global health challenges. The annual growth rate (AGR) of 8.29% in publications highlights the increasing momentum in *B. cereus* research, mirroring the observed shift towards more contemporary approaches. Recent studies have underscored its prevalence in diverse food products, with a global prevalence of 23.75% (Rahnama et al., 2023). Numerous outbreaks, including the well-known “fried rice syndrome,” have been associated with emetic toxin cereulide, highlighting its stability, resistance to food processing, and serious clinical implications (Leong et al., 2023) In addition, infants and young children have been identified as particularly vulnerable populations, often suffering more severe health outcomes such as liver failure and metabolic acidosis from cereulide-producing strains (Li et al., 2025). Early research concentrated heavily on understanding the bacterium’s taxonomy, pathogenic mechanisms, and its role in foodborne illnesses (Dufrenne et al., 1994; Jensen et al., 2003; Kotiranta et al., 2000). More recent works placed instead a greater emphasis on molecular and genomic tools, particularly in relation to understanding the virulence genes, antimicrobial resistance, and the bacterium’s behavior in diverse food matrices (Bianco et al., 2021; Carroll et al., 2022; Tong et al., 2024). For instance, Enosi Tuipulotu et al. (2021) demonstrated the potential of genomic tools to predict toxin production under varying environmental conditions.

These advances reflect the increasing recognition of *B. cereus* as a significant foodborne pathogen, and the need to address the complex challenges it presents in modern food systems. Furthermore, the Document Average Age (DAA) of 6.95 years indicates a healthy balance between recent and established literature.

The Price’s index, calculated at 19.55%, indicates that less than one-fifth of the references in the analyzed literature on *Bacillus cereus* and food safety are from the last 5 years. This relatively low index suggests a slow ageing of references, which carries both positive and negative implications for the field. On the positive side, a reliance on older literature often reflects the foundational nature of earlier studies, which have established the core knowledge base of the discipline. This strong theoretical underpinning ensures that newer research builds upon well-established principles, promoting continuity and conceptual coherence. However, a low Price’s index may also be indicative of stagnation or a slower pace of innovation. When researchers predominantly cite older studies, it may signal limited exploration of emerging trends and an overreliance on traditional frameworks. This can hinder the adoption of cutting-edge methodologies and technologies, potentially slowing the field’s responsiveness to rapidly evolving challenges in food safety. To sustain progress, it is essential to strike a balance between foundational studies and contemporary advancements. Researchers should aim to integrate older, seminal works with recent findings to ensure continuity while advancing the field. Increasing citations of studies published within the last 5 years would help reflect current research directions, while interdisciplinary collaborations could introduce new perspectives, methodologies, and tools. The slow ageing of references may also reflect regional disparities in research output. Countries such as China and South Korea have become dominant contributors to the field, yet their publications often continue to build on older literature rather than introducing novel conceptual or methodological frameworks. In contrast, developing nations like Egypt and Thailand remain underrepresented in the literature, potentially limiting the diversity of perspectives and innovation pathways. The cooperation networks analyzed in this study further highlight the global nature of food safety research. International collaborations are crucial in tackling the multifaceted issues associated with foodborne pathogens. The results revealed that while European and Asian countries tend to form tight-knit collaborative groups, with China playing a central role, regions like North America and Middle East Asia also contribute significantly to global research efforts. These interconnected networks underscore the importance of cross-border cooperation, enabling the sharing of knowledge and resources to address the public health threats posed by *B. cereus*. At researcher level, collaboration patterns revealed distinct clusters formed around specific scientific themes. Some researchers maintain a broad collaborative reach, while others focus more narrowly on niche areas, contributing to specialized knowledge. The analysis further showed how research priorities have evolved over the time, with newer topics such as antimicrobial resistance and the application of genomics gaining prominence in recent years. Additionally, the antimicrobial potential of *B. cereus sensu lato* members—though less appreciated—has emerged as a promising area of research, especially in light of increasing antibiotic and pesticide resistance (Morandini et al., 2024). These biocontrol properties could potentially be leveraged for beneficial applications, further diversifying the role of *B. cereus* beyond pathogenicity.

These trends reflect broader shifts in the scientific community towards a more integrated approach, where interdisciplinarity involving microbiology, food science, and public health plays a central role in shaping future directions. Recent studies have demonstrated how genomic tools are being used to enhance our understanding of *B. cereus* virulence mechanisms. Carroll et al. (2022) utilized genomic sequencing to characterize the *B. cereus* group and identify genes associated with antimicrobial resistance. These findings not only provide critical insights into the genetic determinants of pathogenicity but are also being applied to develop more effective guidelines for managing food safety at the public health level. Another significant example is the study by Bianco et al. (2021), which employed whole-genome sequencing to analyze *B. cereus* isolates from human bacteremia cases. This interdisciplinary approach, combining genomics with clinical microbiology, enabled the identification of specific genetic markers associated with pathogenicity, which can be used to develop rapid diagnostic tools for detecting *B. cereus* contamination in food products. Furthermore, the integration of genomics with public health is evident in efforts to monitor and predict the behavior of *B. cereus* under varying environmental conditions. For example, Tong et al. (2024) combined genomic techniques with epidemiological analyses to assess the prevalence and pathogenicity of *B. cereus* globally. These studies provide essential data for public health agencies, enabling them to implement targeted preventive measures and reduce the risk of contamination throughout the food supply chain.

The keyword “co-occurrence analysis” provided additional insight into the main themes within the field. Terms related to *Bacillus cereus*’s pathogenicity, such as “enterotoxin,” “virulence genes,” and “cereulide,” remain central to the research agenda. However, there has been an important rise in keywords associated with alternative and sustainable control measures, such as “probiotics,” “lactic acid bacteria,” and “antimicrobial resistance.” These trends focus on mitigating contamination risks through natural solutions and on understanding how *B. cereus* adapts to changing food environments, such as plant-based foods and novel preservation techniques. Despite these advancements, there are notable gaps in literature. One of these is the need for further research into the topic of *B. cereus* behavior in emerging food technologies and novel foods, including those arising from the growing plant-based market. In fact, while much attention has been given to traditional food matrices like dairy and meat, emerging food products like plant-based dairy alternatives and processed plant-based proteins present new opportunities and challenges for microbial risk assessment. Additionally, more research is needed to understand the effects of various food processing techniques, such as high-pressure and pulsed electric fields, on the survival of *B. cereus* in these novel products. Another underexplored area is the environmental persistence of *B. cereus* in complex food systems, particularly in large-scale industrial food processing environments. Given the bacterium’s resilience and ability to form spores, it is critical to investigate its survival strategies in diverse environmental conditions and its potential to contaminate food along different stages of the supply chain. Understanding how *B. cereus* interacts with other microorganisms in mixed cultures, as well as its ability to form biofilms, could provide valuable insights into contamination control measures. Further, there is a pressing need for more studies integrating advanced genomic, transcriptomic, and proteomic tools to monitor and predict *B. cereus* behavior under variable conditions. Understanding the genetic determinants of its virulence, especially under different environmental stressors, will be crucial for developing more targeted and effective strategies to prevent foodborne outbreaks. Research in these areas could also help develop rapid diagnostic tools for detecting *B. cereus* contamination in food products. Lastly, interdisciplinary research bridging microbiology, food science, and public health is needed to assess the cumulative impact of *B. cereus* across global food supply chains. Such research should also focus on exploring the effectiveness of alternative control measures, such as natural antimicrobials and innovative food safety interventions, in controlling the bacterium in diverse food systems. Recent works have proposed lactic acid bacteria and probiotics as potential natural inhibitors of *B. cereus* growth and toxin production (Ahmed et al., 2023), underscoring a shift toward biocontrol strategies in food protection. Research on the potential of probiotics and bacteriocins to prevent the growth of *B. cereus* in food matrices could have significant implications for enhancing the protection for consumers without relying solely on chemical preservatives. In conclusion, the findings of this study underscore the dynamic and evolving nature of *B. cereus* research. The scientific community is increasingly leveraging interdisciplinary approaches to address the multifaceted challenges posed by this pathogen in food safety. The development of more sustainable and effective control measures, coupled with the integration of advanced scientific tools, will be key in safeguarding public health in the years to come. While substantial progress has been made, there is still much to learn, particularly regarding the behavior of *B. cereus* in emerging food products and in response to novel preservation technologies. Bridging the gaps in current research will not only deepen our understanding of this bacterium, but also contribute to the development of innovative strategies for mitigating its risks in the global food supply chain. While the search query ‘*Bacillus cereus*’ OR ‘*B. cereus*’ AND ‘Food Safety’ was chosen for its comprehensive relevance to the study’s objectives, we acknowledge that related terms such as ‘foodborne,’ ‘toxin,’ or ‘pathogenicity’ could potentially identify additional relevant studies. However, the selected query aimed to balance specificity with inclusivity, ensuring that retrieved articles were directly aligned with the core themes of *B. cereus* and food safety. Future bibliometric analyses could incorporate broader keyword combinations to capture a wider range of studies addressing related topics, such as toxin production or foodborne outbreaks caused by *B. cereus.*

## Data Availability

The raw data supporting the conclusions of this article will be made available by the authors, without undue reservation.

## Footnotes

^1^ https://www.scopus.com
